# The complex relationship between treatment burden of multimorbidity and self-care in multimorbid patients with hypertension

**DOI:** 10.1186/s12875-025-02916-9

**Published:** 2025-07-09

**Authors:** Kyoung Suk Lee, Jihyang Lee

**Affiliations:** 1 The Research Institute of Nursing Science, College of Nursing, Seoul National University, 103 Daehak-ro, Jongno-gu, Seoul, 03080 South Korea; 2 College of Nursing, Seoul National University, 103 Daehak-ro, Jongno-gu, Seoul, 03080 South Korea

**Keywords:** Multimorbidity, Self-care, Treatment burden, Hypertension

## Abstract

**Background:**

Multimorbid patients with hypertension experienced treatment burden from managing multiple chronic conditions. Although treatment burden can adversely affect self-care, several qualitative studies have suggested a complex relationship between the two factors. This study aimed to identify patient groups based on the level of multimorbidity treatment burden and self-care adherence and explore factors associated with these patient groups. We also examined if patients transitioned to a different group over six months and which factors were associated with either transitioning into or remaining in the ideal group (Lower burden with higher self-care) at six months.

**Methods:**

This longitudinal study included hypertensive patients with at least two comorbidities (*n* = 484); 302 participants completed the 6-month follow-up. Patients were categorized into four groups based on multimorbidity treatment burden and self-care adherence levels: All-low (13.8%); Lower burden with higher self-care (26.0%); Higher burden with lower self-care (35.3%); and All-high (24.8%) groups. Multinomial logistic regression was used to explore factors associated with group membership, with the Lower burden with higher self-care group as the reference group. Binary logistic regression was used to explore factors associated with transitioning into or remaining in the ideal group at six months.

**Results:**

Older age, higher levels of health literacy, better subjective cognitive function, and greater shared decision-making decreased the likelihood of being in the All-low group. Lower depressive symptoms and higher subjective cognitive function decreased the likelihood of being in both Higher burden with lower self-care and All-high groups, while older age and greater shared decision-making were only associated with the Higher burden with lower self-care group. Patients in the All-low and All-high groups frequently transitioned to another group over six months, while the other two groups remained stable. At six months, participants who were male and had higher health literacy, better subjective cognitive function, and greater involvement in shared decision-making were more likely to belong to the ideal group.

**Conclusions:**

Our study observed the complex relationship between multimorbidity treatment burden and self-care adherence in multimorbid patients with hypertension. Interventions aimed at improving shared decision-making considering patients’ circumstances (e.g., emotional status) may alleviate treatment burden and enhance self-care adherence.

**Supplementary Information:**

The online version contains supplementary material available at 10.1186/s12875-025-02916-9.

## Background

Hypertension is a highly prevalent chronic condition globally [1], with significant rates also observed in South Korea [2]. Poorly controlled hypertension including non-adherence to self-care activities can lead to the development of other chronic conditions such as kidney disease and stroke [1]. Numerous studies have shown that patients with hypertension often have at least one additional comorbidity [3, 4], and that hypertension is one of the most common conditions in patients with multimorbidity [5]. Patients with hypertension who have additional chronic conditions experience unique challenges of performing recommended self-care activities compared to patients with only hypertension because of the complex nature of simultaneously managing multiple health conditions [6, 7]. For example, patients with hypertension and comorbidity reported difficulty scheduling multiple medications, concerns about potential interactions between medications, and challenges due to incompatible self-care regimens [6]. Thus, patients with multiple chronic conditions, including multimorbid patients with hypertension, experience the burden of managing multiple self-care regimens and the impacts of the self-care activities imposed on their lives [8]. This is referred to as treatment burden of multimorbidity [9, 10].

Treatment burden of multimorbidity, one of the core patient-reported outcome for patients with multimorbidity [11], is closely related to adherence to self-care [12]. The negative relationship between multimorbidity treatment burden and self-care adherence has been reported in several studies examining patients with multiple chronic conditions [6, 9, 13–17]. While most evidence of this treatment burden for patients with multimorbidity comes from qualitative studies [6, 13–16], several quantitative studies have also shown that greater treatment burden is associated with poorer self-care adherence in patients with chronic conditions including patients with HIV and stroke [18–20].

Several qualitative studies have identified a nuanced relationship in patients with multimorbidity [13–16]. For example, studies have found that some patients intentionally do not adhere to the recommended healthcare tasks to alleviate their treatment burden [13–15], whereas some patients faithfully perform the recommended self-care activities despite the significant treatment burden [15, 16]. The results of these qualitative studies highlight the intricate relationship between treatment burden and self-care, underscoring the importance of considering both factors simultaneously to fully understand the relationship.

The purpose of this study was to explore factors associated with patient groups categorized by the levels of multimorbidity treatment burden and self-care adherence among multimorbid patients with hypertension. Specific aims were (1) to identify distinct patient groups based on the levels of multimorbidity treatment burden and self-care adherence and to describe the characteristics of each group and (2) to explore factors associated with these patient groups. Treatment burden and self-care adherence change over time, and there is often a cyclical pattern between the two factors [12, 21]. For example, an increase in treatment burden can lead to non-adherence to self-care, which, in turn, may further elevate treatment burden [12, 21]. Therefore, we conducted a 6-month follow-up to examine if patients transitioned to another group from baseline to follow-up and which factors were associated with either transitioning into or remaining in the ideal group (i.e., Lower burden with higher self-care) at six months.

## Methods

### Study design and participants

This longitudinal observational study recruited patients with hypertension and comorbidities from a large online platform. Participants were followed up at six months after the baseline assessment. Eligible patients in this study were individuals who were older than 19 years, had been diagnosed or currently being treated with hypertension at least three months before enrollment, and had at least two additional chronic conditions. Exclusion criteria included having a diagnosis of dementia or unable to read Korean. Participants’ comorbidities were assessed using a list of self-reported chronic diseases developed by Fortin and colleagues [22] (Supplementary Table 1). Participants were asked whether they had been diagnosed by a clinician and/or were currently being treated for any of the listed conditions.

### Procedures

This study was approved by the Institutional Review Board at Seoul National University (IRB No. 2306/001–012) and complied with the Declaration of Helsinki. The study invitation emails or text messages with a link to the information about the survey were sent to individuals registered on an online research panel platform where people participate in surveys of their interest. This panel consists of 1,752,795 people (about 3.4% of the Korean population, 56% female, aged 18 to 80 years) residing across Korea. They voluntarily participated in the study after reading an invitation email containing an explanation of the study. Individuals who were interested in our study completed a set of screening questions to confirm that they met the eligibility criteria (e.g., age, diagnosis of at least two chronic conditions and hypertension). Those who met the eligibility criteria provided signed, informed consent and completed structured questionnaires through self-report. Six months after the baseline survey, a follow-up survey link was sent to the participants who completed the baseline assessment.

### Measures

To explore factors associated with patient groups categorized by the levels of multimorbidity treatment burden and self-care adherence, we collected baseline measures to assess multimorbidity treatment burden and self-care adherence. We also assessed additional factors that have been consistently reported in the literature as correlates of multimorbidity treatment burden and self-care adherence (i.e., health literacy, subjective cognitive function, depressive symptoms, and shared decision-making) [12, 13, 23–29]. We also collected self-reported socio-demographic and clinical information at baseline. To examine whether patients transitioned to another group from baseline to the 6-month follow-up, we conducted a 6-month follow-up survey that included measures for multimorbidity treatment burden, self-care, disease burden, and depressive symptoms that were expected to change over time.

#### Multimorbidity treatment burden

The treatment burden of multimorbidity was measured with the 13-item Multimorbidity Treatment Burden Questionnaire (MTBQ) [28]. Each item was rated on a 5-point Likert scale. Total scores range from 0 to 100, with higher scores indicating a greater multimorbidity treatment burden. Patients were categorized into four burden groups based on the total scores: No burden (score = 0), Low burden (score < 10), Medium burden (score 10–22), and High burden (score > 22) [28]. The psychometric soundness of both the English and Korean versions have been reported in previous studies [28, 30, 31].

#### Self-care

Overall self-care of chronic conditions was measured with the Partners In Health Scale (PIH) [32, 33]. The 12-item PIH is a generic instrument measuring self-care behaviors of patients with chronic conditions [33]. Each item was rated on a 9-point Likert scale. Total scores range from 0 to 88, with higher scores indicating better self-care adherence. Currently, no established cut-off points exist for the PIH [32, 33]. The validity of the Korean version of the PIH was supported [34].

#### Health literacy

The Newest Vital Sign (NVS) instrument was used to measure patients’ health literacy [35]. The NVS consists of an ice-cream nutrition label accompanied by six open-ended questions. Correct answers to the questions are summed for a total score ranging from 0 to 6 [35]. The Korean version of the Newest Vital Sign showed good psychometric properties [36].

#### Subjective cognitive function

The Attentional Function Index was used to measure patients’ perceived levels of effectiveness in planning and engaging in everyday activities requiring attention and working memory [37]. Patients were asked to rate 16 items on an 11-point Likert scale. Total scores range from 0 to 10, with higher scores indicating better perceived cognitive functioning. Psychometric testing of the English and Korean versions was satisfactory [37, 38].

#### Depressive symptoms

Depressive symptoms were measured with the 9-item Patient Health Questionnaire [39, 40]. The nine items correspond to the diagnostic criteria for major depressive disorders in the Diagnostic and Statistical Manual of Mental Disorder IV. Each item was rated on a 4-point Likert scale ranging from 0 to 3. Total scores range from 0 to 27, with higher scores indicating higher levels of depressive symptoms. Scores of 10 and higher indicate clinically significant depressive symptoms [39].

#### Shared decision-making

Shared decision-making was measured with the collaboRATE instrument [41]. This 3-item self-reported instrument asks patients to rate the extent to which their healthcare providers have attempted to incorporate shared decision-making in their clinical encounters, using a 5-point Likert scale ranging from 0 to 4. Total scores were calculated as the average of the sum of the responses to each item, with higher scores indicating a greater degree of shared decision-making. The Korean version of the instrument showed good psychometric properties [42].

#### Socio-demographic and clinical information

Patients were asked to provide self-reported socio-demographic and hypertension-related information. We measured disease burden by adapting the Disease Burden Impact Scale [43], using the list of chronic conditions developed by Fortin and colleagues [22]. This list included 20 chronic conditions with a free text option for any additional condition. Patients were first asked about the presence of diagnosed and/or treated chronic conditions. If they reported having one or more chronic condition, they were then asked to rate the level of interference with daily activities due to each of those conditions on a 5-point Likert scale ranging from 1 to 5. Total scores were derived by summing the responses, with higher scores indicating higher levels of disease burden.

### Statistical analyses

Patients were categorized based on the scores of multimorbidity treatment burden and self-care using the cutoff values of the MTBQ (< 10 for no to low burden vs. ≥ 10 for medium to high burden) and median values of the PIH (< 68 vs. ≥ 68): (1) lower burden and lower self-care adherence (All-low group); (2) lower burden and higher self-care adherence (Lower burden with higher self-care group); (3) higher burden and lower self-care adherence (Higher burden with lower self-care group); and (4) higher burden and higher self-care adherence (All-high group). Because no established cutoff value exists for the PIH, we used the median value to classify participants into lower or higher self-care groups.

Group comparisons among the four patient groups were conducted with one-way analysis of variance (ANOVA) or chi-square tests, as appropriate. If F-statistics of ANOVA were significant, a Scheffe’s test was used to determine group differences. For disease burden scores, PHQ scores and MTBQ scores at baseline and six months, as well as PIH scores at baseline, Welch’s ANOVA was used when the assumption of equal variances was not met. This method adjusts for unequal variances and was followed by Games-Howell post hoc tests for pairwise comparisons.

Multinomial logistic regression analysis was conducted to determine the factors associated with the four patient groups. Variable selection for the multinomial logistic regression was guided by an integrated framework and literature review on multimorbidity treatment burden and self-care adherence [12, 13, 23–29]. To avoid overfitting and ensure conceptual relevance, we prioritized variables associated with both multimorbidity treatment burden and self-care in chronic condition, rather than disease-specific indicators. Prior to model estimation, multicollinearity using variance inflation factors (VIFs) was assessed, and no issues were found with the VIFs ranging from 1.06 to 1.21. Lasagna plots [44] were used to visualize the patient group transition between the baseline and the six-month follow-up. We also conducted a binary logistic regression to explore factors of being in the ideal group (i.e., Lower burden with higher self-care group) at six months through transition or sustained status. Data were analyzed with IBM SPSS version 26.

## Results

### Sample characteristics

The mean age of the sample (*N* = 484), was 53 years (SD 10.2). The majority of patients were men (76.0%) and lived with someone (87.8%) (Table 1). Patients had an average of 3.2 comorbidities, with the most prevalent comorbidity of hyperlipidemia (69.2%) followed by obesity (59.3%) and diabetes (43.0%). Clinically significant depressive symptoms (score of ≥ 10) at baseline were found in 26.0% of the patients.

**Table 1:** Sample characteristics (*n* = 484)

Characteristics	All subjects (*n* = 484)	All-low (*n* = 67)	Lower burden with higher self-care (*n* = 126)	Higher burden with lower self-care (*n* = 171)	All-high (*n* = 120)	*p*-value
	*n* (%) or mean (SD)					
Age, year	53.3 (10.2)	52.8 (10.9)^bc^	56.8 (8.6)^a^	50.0 (10.0)^c^	54.5 (10.3)^ab^	< 0.001
Sex						0.149
Male	368 (76.0%)	49 (73.1%)	88 (69.8%)	133 (77.8%)	98 (81.7%)	
Female	116 (24.0%)	18 (26.9%)	38 (30.2%)	38 (22.2%)	22 (18.3%)	
Living with someone	425 (87.8%)	55 (82.1%)	119 (94.4%)	143 (83.6%)	108 (90.0%)	0.014
Education level						0.584
< high school graduation	2 (0.4%)	0 (0.0%)	1 (0.8%)	0 (0.0%)	1 (0.8%)	
≥ high school graduation	482 (99.6%)	67 (100.0%)	125 (99.2%)	171 (100.0%)	119 (99.2%)	
Employment status						0.086
Full or part-time	361 (74.6%)	48 (71.6%)	91 (72.2%)	139 (81.3%)	83 (69.2%)	
Unemployed	123 (25.4%)	19 (28.4%)	35 (27.8%)	32 (18.7%)	37 (30.8%)	
Perceived economic status	2.7 (0.9)	2.6 (0.9)	2.8 (0.8)	2.5 (0.9)	2.8 (0.9)	0.011
Years since hypertension diagnosis	8.6 (7.1)	8.5 (8.5)	8.7 (6.8)	7.5 (5.9)	10.0 (7.8)	0.029
Recent blood pressure reading of < 140/90mmHg	229 (55.7%)	39 (66.1%)	74 (63.8%)	60 (45.8%)	56 (53.3%)	0.011
Taking ≥ 5 medications a day	60 (19.9%)	7 (15.6%)	16 (16.8%)	22 (19.1%)	15 (32.6%)	0.122
Number of comorbidities	4.2 (1.3)	4.2 (1.2)	4.1 (1.2)	4.2 (1.4)	4.3 (1.4)	0.603
Disease burden	11.0 (6.3)	9.8 (6.1)^bc^	9.0 (5.0)^c^	11.9 (6.2)^ab^	12.4 (7.3)^a^	< 0.001^§^
Disease burden after 6 months (*n* = 302)	11.5 (6.9)	10.3 (8.0)^ab^	9.4 (4.8)^b^	12.5 (6.6)^a^	13.0 (7.7)^a^	< 0.001^§^
Health literacy	2.9 (1.6)	2.6 (1.6)	3.0 (1.6)	3.1 (1.7)	2.7 (1.6)	0.062
Subjective cognitive function	6.5 (1.6)	6.5 (1.3)^b^	7.8 (1.4)^a^	5.6 (1.5)^c^	6.5 (1.3)^b^	< 0.001
Depressive symptoms	6.6 (5.7)	4.4 (4.8)^b^	3.6 (4.1)^b^	8.9 (5.8)^a^	7.7 (5.8)^a^	< 0.001^§^
Depressive symptoms after 6 months (*n* = 302)	6.2 (5.7)	4.0 (4.4)^b^	3.7 (4.5)^b^	8.3 (5.9)^a^	7.4 (5.9)^a^	< 0.001^§^
Shared decision-making	1.6 (0.9)	1.4 (0.8)^b^	1.9 (1.0)^a^	1.3 (0.8)^b^	1.8 (0.9)^a^	< 0.001
Multimorbidity treatment burden	20.5 (20.4)	4.1 (3.6)^b^	3.3 (3.2)^b^	33.0 (19.1)^a^	30.0 (19.4)^a^	< 0.001^§^
Multimorbidity treatment burden after 6 months (*n* = 302)	17.7 (17.4)	7.9 (7.8)^b^	5.3 (7.6)^b^	26.9 (16.1)^a^	25.1 (19.7)^a^	< 0.001^§^
Self-care	66.9 (15.4)	57.1 (8.7)^c^	81.8 (7.0)^a^	52.6 (10.2)^d^	77.1 (6.0)^b^	< 0.001^§^
Self-care after 6 months (*n* = 302)	67.2 (14.6)	62.1 (12.7)^c^	79.3 (9.5)^a^	57.1 (12.2)^c^	69.8 (12.6)^b^	< 0.001

Perceived economic levels measured using a five-point rating scale (1 = far below average, 3 = average, 5 = far above average). Higher scores on health literacy, subjective cognitive function, depressive symptoms, and shared decision-making indicate better health literacy, better subjective cognitive function, more severe depressive symptoms, and greater involvement in shared decision-making, respectively. Different superscript letters in the same row represent significantly different values for each variable (*p* < 0.05) based on Scheffe’s post hoc test unless indicated otherwise. §= Disease burden, depressive symptoms, multimorbidity treatment burden scores at baseline and after 6 months, as well as self-care scores at baseline were analyzed with Welch’s ANOVA, followed by Games-Howell post hoc tests. Values are baseline data unless indicated as 6-month data

Of the 484 participants, 302 (62.4%) completed the follow-up at six months and reported having a medium level of multimorbidity treatment burden, on average. There were no significant differences in the baseline characteristics and levels of multimorbidity treatment burden and self-care adherence between patients who completed the follow-up survey and those who did not.

### Patient groups and sample characteristic comparison

Based on scores of multimorbidity treatment burden and self-care adherence, patients were categorized into four groups: 13.8% (*n* = 67) in the All-low (i.e., Lower burden with lower self-care); 26.0% (*n* = 126) in the Lower burden with higher self-care; 35.3% (*n* = 171) in the Higher burden with lower self-care; and 24.8% (*n* = 120) in the All-high (i.e., Higher burden with higher self-care) (Table 1).

When comparing the differences among the four groups in demographic and clinical characteristics at baseline, significant group differences were found in age, living arrangement, levels of perceived economic status, hypertension duration, and blood pressure reading (*p*-values < 0.05). However, no significant differences were found in sex, education level, employment status, taking more than five medications, and the number of comorbidities (Table 1). Patients in the Lower burden with higher self-care group were more likely to have higher levels of subjective cognitive function compared to the other patient groups. Compared to patients in the Lower burden with higher self-care group, those in the Higher burden with lower self-care and All-high groups reported higher levels of disease burden and depressive symptoms (*p*-values < 0.001). Levels of shared decision-making between the Lower burden with higher self-care group and All-high group were not significantly different but were greater than those observed in the other two groups (*p*-values < 0.001).

When comparing variables measured at six months, patients in the Lower burden with higher self-care group were more likely to have lower levels of disease burden compared to those in the Higher burden with lower self-care and All-high groups (*p*-value < 0.001). Higher levels of depressive symptoms were observed in patients in the Higher burden with lower self-care and All-high groups at the 6-month follow-up than those in the other groups, which were similar to the baseline findings (*p*-value < 0.001).

### Factors associated with group memberships

A multinomial logistic regression model was used to determine factors associated with each patient group (Table 2). Older age (adjusted odds ratio [aOR] = 0.964), higher levels of health literacy (aOR = 0.763), better subjective cognitive function (aOR = 0.478), and a greater degree of shared decision-making (aOR = 0.440) were associated with a decreased likelihood of being in the All-low group compared to the Lower burden with higher self-care group.

**Table 2:** Results of multivariate multinomial logistic regression (*n* = 484)

Variables	All-low^a^		Higher burden with lower self-care^a^		All-high^a^	
	OR (95% CI)	*p*-value	OR (95% CI)	*p*-value	OR (95% CI)	*p*-value
Age	0.964 (0.929-1.000)	0.049	0.961 (0.930–0.993)	0.018	1.004 (0.971–1.038)	0.816
Male	1.275 (0.600-2.712)	0.527	1.490 (0.756–2.936)	0.250	1.838 (0.937–3.608)	0.077
Living with someone	0.459 (0.144–1.460)	0.187	0.701 (0.234-2.100)	0.526	0.843 (0.271–2.627)	0.769
Perceived economic status	0.942 (0.628–1.413)	0.772	1.182 (0.821-1.700)	0.369	1.208 (0.850–1.717)	0.293
Disease burden	1.002 (0.937–1.071)	0.962	1.022 (0.967–1.080)	0.444	1.034 (0.983–1.089)	0.193
Health literacy	0.763 (0.617–0.943)	0.012	0.919 (0.760–1.111)	0.381	0.871 (0.725–1.048)	0.143
Subjective cognitive function	0.478 (0.358–0.637)	< 0.001	0.370 (0.284–0.482)	< 0.001	0.566 (0.442–0.725)	< 0.001
Depressive symptoms	0.945 (0.863–1.035)	0.225	1.097 (1.018–1.181)	0.015	1.109 (1.031–1.192)	0.005
Shared decision-making	0.440 (0.293–0.661)	< 0.001	0.341 (0.236–0.493)	< 0.001	0.762 (0.550–1.055)	0.102
Likelihood ratio *χ*^2^ (*df*) = 259.020 (27), *p* < 0.001						

^a^Reference category: Lower burden with higher self-care group, *CI *Confidence Interval. Higher scores on health literacy, subjective cognitive function, depressive symptoms, and shared decision-making indicate better health literacy, better subjective cognitive function, more severe depressive symptoms, and greater involvement in shared decision-making, respectively

Patients in the Higher burden with lower self-care group were more likely to be younger (aOR = 0.961) and experience lower levels of subjective cognitive function (aOR = 0.370), greater depressive symptoms (aOR = 1.097), and a lower degree of shared decision-making (aOR = 0.341) compared to those in the Lower burden with higher self-care group. Having greater depressive symptoms (aOR = 1.109) with lower levels of subjective cognitive function (aOR = 0.566) was associated with an increased likelihood of being in the All-high group compared to the Lower burden with higher self-care group.

### Patient group transition after six months

**Fig. 1:**
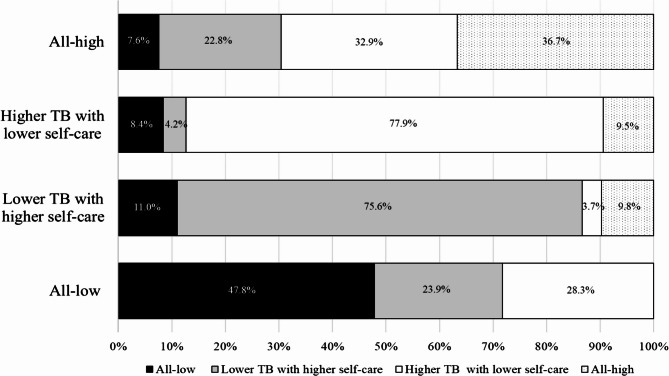
Transition of patient groups by levels of multimorbidity treatment burden and self-care over six months Note. TB: Treatment burden; The percentage of patients who transitioned at six monthswas shown by the % values in each bar

A majority of the patients in the Lower burden with higher self-care group (75.6%) and the Higher burden with lower self-care group (77.9%) stayed in their respective groups over six months, while fewer than half of the patients in the other two groups remained the same (47.8% All-low and 36.7% All-high groups) (Fig. 1). Within the All-low group at baseline, 23.9% and 28.3% moved to the Lower burden with higher self-care group and the Higher burden with lower self-care group, respectively. In the All-high group at baseline, 22.8% and 32.9% moved to the Lower burden with higher self-care group and the Higher burden with lower self-care group, respectively.

We also explored which factors were associated with being in the ideal group (i.e., the Lower burden with higher self-care group) at six months (Table 3). Participants who either remained in or transitioned to the ideal group at six months were more likely to be male compared to those in all other groups at six months (aOR = 2.484). Higher levels of health literacy (aOR = 1.214), better subjective cognitive function (aOR = 1.876), and a higher degree of shared decision-making (aOR = 1.852) were also associated with a greater likelihood of belonging to the ideal group at six months.

**Table 3:** Factors associated with being in Lower burden with higher self-care group at 6-month follow-up (*n* = 302)

Variables	Odds Ratio (95% Confidence interval)	*p*-value
Age	1.035 (1.000-1.072)	0.053
Male	2.484 (1.233–5.006)	0.011
Living with someone	3.116 (0.912–10.638)	0.070
Perceived economic status	0.749 (0.520–1.078)	0.120
Change in disease burden^§^	0.950 (0.885–1.019)	0.151
Health literacy	1.214 (1.005–1.466)	0.044
Subjective cognitive function	1.876 (1.482–2.376)	< 0.001
Change in depressive symptoms^§^	0.996 (0.916–1.084)	0.934
Shared decision-making	1.852 (1.292–2.655)	0.001

Reference category: Patients who were in groups other than the Lower burden with higher self-care group at 6 months. Higher scores on health literacy, subjective cognitive function, depressive symptoms, and shared decision-making indicate better health literacy, better subjective cognitive function, more severe depressive symptoms, and greater involvement in shared decision-making, respectively. ^§^ Changes in disease burden and depressive symptoms were calculated by subtracting baseline scores from 6-month scores. Values are baseline data unless indicated as change scores

We conducted an additional analysis to rule out the possibility that group transitions were driven by borderline cases with minimal change (i.e., PIH scores fell within ± 3 points of the median cutoff). Of the 16 patients in the All-high group, 4 remained within the borderline range at follow-up, while the remaining 12 transitioned into other groups with a mean change in PIH scores of − 7.2 points. Similar patterns were observed in the other groups (All-low: 2 of 7; Lower burden with higher self-care: 1 of 6; Higher burden with lower self-care: 2 of 7). These results indicate that only a small proportion of patients had borderline scores at baseline, and most did not remain in that range at follow-up, suggesting that group transitions were not simply due to minor fluctuations around the cutoff.

## Discussion

We identified four distinct groups by the levels of multimorbidity treatment burden and self-care adherence in multimorbid patients with hypertension: All-low, Lower burden with higher self-care, Higher burden with lower self-care, and All-high groups. Our study showed that multimorbidity treatment burden levels remained relatively stable over time, which is in line with previous studies on patients with multimorbidity [9, 45]. However, patients showing a counter-intuitive relationship between multimorbidity treatment burden and self-care (i.e., All-low and All-high groups) often transitioned to other groups, while patients showing typical patterns tended to remain in the same group. We also found that health literacy, subjective cognitive function, depressive symptoms, and shared decision-making differentiated group membership, while higher levels of health literacy, better subjective cognitive function, and greater degree of shared decision-making were associated with being in the Lower burden with higher self-care at six months.

We found a small subset of patients (13.8%) with both lower levels of multimorbidity treatment burden and self-care adherence. Younger age and lower levels of health literacy, poorer subjective cognitive function, and lower degree of shared decision-making increased the likelihood of being in the All-low group compared to the Lower burden with higher self-care group. Higher levels of health literacy and better subjective cognitive function are well-established factors related to greater self-care adherence [14, 25, 27, 46], while active involvement in shared decision-making is a common intervention target to mitigate multimorbidity treatment burden [24, 26].

Although patients in the All-low group experienced difficulty following the recommended self-care, they reported lower levels of multimorbidity treatment burden. This counter-intuitive relationship may be because the All-low group had relatively low levels of disease burden and better controlled blood pressure compared to the two groups with higher burden. These characteristics may make patients in the All-low group put low priority on self-care, which could contribute to non-adherence [47], and a subsequent decrease in multimorbidity treatment burden. However, it is also possible that patients intentionally decide to not follow some recommended self-care activities to alleviate treatment burden, which has been reported in previous studies [13–15]. In one qualitative study, a patient described her intentional non-adherence to asthma medications as “rebellion” to actively decrease her burden of taking medications for multiple conditions [14]. This decision also aligns with findings by Oh and Lee [48] who found that the most common reason hypertensive patients with comorbidities gave for medication non-adherence across multiple conditions was that their conditions were “not severe enough.” Thus, further studies are needed to investigate the nature of non-adherence in this group.

We found that 24.8% of the patients reported higher multimorbidity treatment burden and self-care adherence (i.e., All-high group). Greater depressive symptoms and lower levels of subjective cognitive function were associated with an increased likelihood of belonging to the All-high group compared to those in the Lower burden with higher self-care group, while the levels of shared decision-making were comparable between the two group. Shared decision-making may have helped mitigate the potential negative impacts of cognitive dysfunction and depressive symptoms in the All-high group, which might have contributed to them maintaining a relatively high level of self-care. However, about a third (32.9%) of the participants in the All-high group continued to experience high multimorbidity treatment burden while decreasing adherence to self-care at the 6-month follow-up. This change may increase the risk for poor health outcomes. Therefore, consistent and high-quality shared decision-making may be particularly important to support patients in this group.

Previous studies have shown that patients with chronic conditions who actively took responsibility for their self-care tended to adhere well to recommended self-care activities [49–51]. When patients recognized the benefits of self-care activities including improving health, their efforts to manage their health conditions were perceived as positive experiences, which contributed to a decrease in multimorbidity treatment burden [52]. However, some patients reported feeling burdened or ashamed when their self-care efforts did not lead to improved health [49]. In our study, patients in the All-high group tended to experience higher disease burden and uncontrolled blood pressure compared to those in the Lower burden with higher self-care group. The All-high group may have experienced increased multimorbidity treatment burden due to the lack of a noticeable improvement in their health despite their efforts. Similarly, patients who felt a sense of loss of control over managing their health conditions reported that they felt a higher burden from managing their health [29]. Greater depressive symptoms, which emerged as a distinct factor in the All-high group and Higher burden with lower self-care group, may exacerbate All-high group patients’ burden in performing self-care activities as depressive symptoms are a critical barrier to self-care adherence in patients with chronic conditions, such as affecting motivation and cognitive capacity [53, 54].

Patients in the Higher burden with lower self-care group constituted the largest portion of our sample (35.3%) with younger age, and most patients remained in this group over six months. The factors associated with membership in this group were not widely different from factors found in the All-low and All-high groups. However, this group had the lowest levels of subjective cognitive function among the four groups, which was lower than that of older community dwellers in Korea [55]. Additionally, their levels of depressive symptoms and the rate of uncontrolled blood pressure were higher than those in the All-low group. Their depressive symptoms may have influenced their ability to engage in self-care or control their health status, which may in turn have increased their burden of self-care activities. This group showed little transition over six months, which may indicate the need for active, intensive support to help these patients transition to the Lower burden with higher self-care group.

In our study, health literacy, subjective cognitive function, and shared decision-making were key factors associated with favorable group membership at six months. These same factors also distinguished group membership at baseline. This finding suggests that targeted interventions addressing these domains may help shift patients toward a more favorable trajectory over time. The shared decision-making process has the potential to address both health literacy and cognitive dysfunction by actively involving patients in care planning, clarifying complex health information, and supporting informed, value-congruent choices.

In two studies [24, 26], shared decision-making was implemented to decrease multimorbidity treatment burden, but these studies reported different results. The non-randomized study by Tinetti and colleagues showed positive outcomes by aligning patient care with individual health priorities [26], which involved discussing self-care activities and medical procedures with patients to identify burdensome healthcare tasks. As a result, clinicians were more likely to discontinue medications and were less likely to recommend additional self-care activities for patients in the intervention group compared to the usual care group. In contrast, a cluster-randomized study by Salisbury and colleagues did not show changes in the number of prescribed medications and level of medication adherence and multimorbidity treatment burden after implementing a comprehensive multidisciplinary review and health plan development [24].

These results support the importance of clinicians discussing healthcare tasks that patients perceive as burdensome or as manageable, which could improve adherence to treatment even for patients with limited internal resources (e.g., cognitive function or health literacy). In addition, aligning patients’ care with their health priorities could help patients understand that responsibility for self-care can be shared by clinicians. This approach may help decrease multimorbidity treatment burden, especially in patients in the All-high group who may experience negative emotions when their health status does not improve despite their self-care efforts.

Another possible reason for the different results may be the intensity of the intervention. The Salisbury and colleagues’ study involved only two sessions over 15 months, with only around half of the participants attending both sessions. In particular, this low intensity may be insufficient for patients with higher treatment burden and lower self-care adherence, as indicated by our study showing that most of these patients remained in the same group over six months. Adding interventions to address depressive symptoms could also enhance patient outcomes, as depressive symptoms negatively impacted multimorbidity treatment burden in our study and other studies [13, 16, 28].

Several limitations in this study should be noted. First, the generalizability of our findings is limited due to the use of convenience sampling from an online panel. Although we were able to reach participants across various locations in Korea, the sample may not be fully representative of our population. It is important to note that Korea has high internet accessibility, with an internet usage rate of 88.4% among Koreans in their 50 s, which corresponds to the average age of our sample [56]. However, the mean age of participants in our study (53.1 years) was lower than the median age of 62.6 years observed in studies included in a meta-analysis on the global prevalence of multimorbidity [57]. In addition, our sample was highly educated, with 99.4% of participants having at least a high school education. However, the population in Korea generally has a high level of education, with 89% of the population aged 25 to 64 having attained at least a high school education [58]. The follow-up completion rate at six months was 62.4%, which is slightly lower than the rates reported in previous observation studies [59, 60]. Although no significant differences were found in baseline characteristics and levels of multimorbidity treatment burden and self-care adherence between patients who completed and did not complete follow-up, selection bias cannot be ruled out. Therefore, the results need to be interpreted with caution. Second, the current study relied on self-reports of the number of comorbidities, which may not be accurate. However, the list of comorbidities used in our study was developed to measure self-reported chronic conditions in primary care settings. In addition, because patients are likely to perform self-care for chronic conditions they are aware of, using self-reported measures for chronic conditions aligned with the purpose of our study. The third limitation is the use of a median split method due to the lack of established cut-off points, which may differ from clinically meaningful thresholds. However, a median split method is a commonly used approach in the absence of validated cutoffs and does not inflate the risk of Type I error when predictor variables are uncorrelated [61]. Future studies are needed to establish clinically meaningful thresholds for interpreting PIH scores. Last, this study used an observational design and did not measure changes in variables included in the multinomial logistic regression model over six months, except for disease burden and depressive symptoms. Therefore, our results should be interpreted with caution.

Despite these limitations, our study identified complex and dynamic patterns of patient typologies based on levels of multimorbidity treatment burden and self-care adherence using a longitudinal design. Our findings also identified important factors that were associated with favorable group transitions (e.g., higher levels of health literacy and greater involvement in shared decision-making), which can provide valuable insights to guide the development of tailored interventions.

## Conclusion

Our study explored the complex relationship between multimorbidity treatment burden in patients with hypertension and comorbidities. We identified four distinct groups by the level of multimorbidity treatment burden and self-care adherence and found that these groups were not static. Patients frequently transitioned between groups over six months, especially among patients with counter-intuitive relationships. However, we found that the group with higher treatment burden and lower self-care adherence did not frequently transition to another group. Our study indicates that health literacy, subjective cognitive function, depressive symptoms and shared decision-making are related to group membership and being in the ideal group at six months.

Our study highlights the need to target the group with higher treatment burden and lower self-care adherence with effective interventions to improve health outcomes. Interventions aimed at improving shared decision-making by considering patients’ circumstances (e.g., emotional status and internal resources) may help alleviate multimorbidity treatment burden and enhance self-care adherence. Before developing interventions, it is critical to gain a deeper understanding of the relationship between multimorbidity treatment burden and self-care adherence from patients’ perspective. This result can lead to more effective and tailored strategies to address the unique needs of these patients.

## Supplementary Information

Supplementary Material 1.

## Data Availability

The data underlying this article will be shared upon reasonable request to the corresponding author.
