# Supplementary material for: The complex relationship between treatment burden of multimorbidity and self-care in multimorbid patients with hypertension
Source: BMC Prim Care. 2025 Jul 9;26:219. doi: 10.1186/s12875-025-02916-9 (PMC12239294; doi:10.1186/s12875-025-02916-9)
Supplement: Supplementary file 1 — Supplementary Material 1. [file 12875_2025_2916_MOESM1_ESM.docx]

**Supplementary Table 1. List of chronic conditions (Fortin et al., 2017)**

| Hypertension |
| --- |
| Hyperlipidemia |
| Asthma, chronic obstructive pulmonary disease or chronic bronchitis |
| Thyroid disease |
| Stomach problem (reflux, ulcer of heartburn) |
| Obesity |
| Osteoporosis |
| Heart failure (including valve problems or replacement) |
| Stroke and transient ischemic attack |
| Depression or anxiety |
| Chronic urinary problem |
| Chronic hepatitis |
| Diabetes |
| Chronic musculoskeletal conditions causing pain or limitations |
| Colon problems (irritable bowel, Crohn’s disease, ulcerative colitis, diverticulosis) |
| Arthritis or rheumatoid arthritis |
| Any cancer in the previous 5 years (including melanoma but excluding other skin cancers) |
| Cardiovascular disease (angina, myocardial infarction, atrial fibrillation, poor circulation in the lower limbs) |
| Kidney disease or failure |
| Others |

**Reference**

Fortin, M., Almirall, J., & Nicholson, K. (2017). Development of a research tool to document self-reported chronic conditions in primary care. *J Comorb*, *7*(1), 117-123. <https://doi.org/10.15256/joc.2017.7.122>
